# Transient phrenic nerve stunning during superior vena cava isolation after focal pulsed field ablation

**DOI:** 10.1016/j.hrcr.2026.04.018

**Published:** 2026-04-20

**Authors:** Kohei Ukita, Roman Mamaev, Charlotte Eitel, Roland Richard Tilz

**Affiliations:** 1 Department of Rhythmology, University Heart Center Lübeck, University Hospital Schleswig-Holstein, Lübeck, Germany; 2 German Center for Cardiovascular Research, Partner Site Lübeck, Germany

**Keywords:** Atrial fibrillation, Catheter ablation, Phrenic nerve stunning, Pulsed field ablation, Superior vena cava

**Key Teaching Points**

• Although pulsed field ablation (PFA) is generally considered to have a favorable safety profile with respect to phrenic nerve injury, transient phrenic nerve stunning can occur during superior vena cava (SVC) isolation using a focal PFA catheter.
• The rapid and complete phrenic nerve recovery observed in this case supports a functional rather than structural nerve injury, consistent with transient neural stunning rather than permanent phrenic nerve palsy.
• This case also highlights the importance of procedural vigilance during SVC ablation using PFA, including phrenic nerve pacing, continuous monitoring of diaphragmatic movement, and prompt interruption of energy delivery if diaphragmatic weakening is observed.

## Introduction

Pulsed field ablation (PFA) has emerged as a nonthermal ablation modality that induces irreversible electroporation of cardiomyocytes while relatively sparing surrounding noncardiac tissues, including the esophagus and phrenic nerve.^1^ Owing to its myocardial selectivity, PFA is increasingly used for pulmonary vein isolation (PVI) and adjunctive ablation strategies in patients with atrial fibrillation (AF).

The superior vena cava (SVC) has been recognized as 1 of the non–pulmonary vein foci contributing to the initiation and maintenance of AF.^2^^,^^3^ However, SVC isolation carries an inherent risk of phrenic nerve injury owing to close anatomic proximity. Although PFA is generally considered to have a favorable safety profile with respect to phrenic nerve injury, both transient and persistent phrenic nerve dysfunction have been reported in association with PFA procedures.^4^, ^5^, ^6^, ^7^, ^8^, ^9^

Recently, a novel dual-energy, flexible-tip focal ablation catheter capable of transmitting either radiofrequency energy or PFA therapy within a single device has been introduced into clinical practice. In this report, we describe a case of transient phrenic nerve stunning observed during SVC isolation using the PFA mode of a novel dual-energy, focal ablation catheter.

## Case report

**Figure 1:**
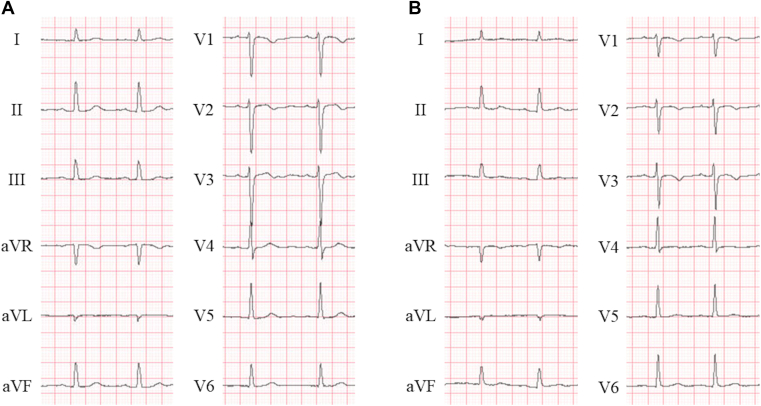
**A:** A 12-lead electrocardiogram before the repeat ablation procedure showing sinus rhythm (25 mm/s, 10 mm/mV). The heart rate was 67 beats per minute. **B:** A 12-lead electrocardiogram after the repeat ablation procedure demonstrating sinus rhythm (25 mm/s, 10 mm/mV). The heart rate was 77 beats per minute.

A 77-year-old woman with symptomatic recurrent persistent AF was referred for repeat catheter ablation. The patient had previously undergone PVI, left atrial posterior wall isolation, left atrial anterior line ablation, cavotricuspid isthmus ablation, and left atrial appendage occluder implantation (WATCHMAN FLX, Boston Scientific). A 12-lead electrocardiogram before the repeat ablation procedure revealed sinus rhythm (Figure 1A). Laboratory tests showed a normal N-terminal pro-B-type natriuretic peptide level of 100 pg/mL. Echocardiography demonstrated normal left ventricular function and a normal-sized left atrium.

**Figure 2:**
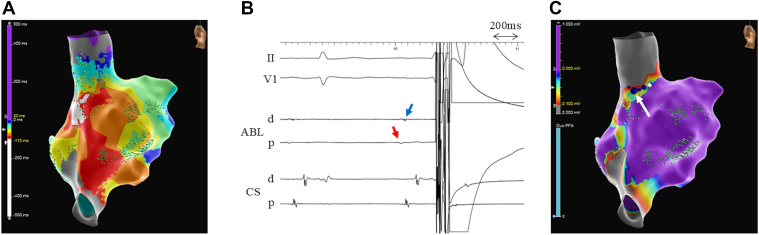
**A:** An activation map of the right atrium (RA) and superior vena cava (SVC) obtained with the HD Grid (right lateral view). The sinus node region was identified as the *white area*. **B:** An intracardiac electrocardiogram when applications were delivered using the TactiFlex Duo. The RA potential was observed at the proximal electrode (*red arrow*) and the SVC potential at the distal electrode (*blue arrow*) before application. **C:** A voltage map obtained with the HD Grid after SVC isolation and the application tags (right lateral view). The tag indicated by the *white arrow* represents the application associated with transient phrenic nerve stunning. ABL = ablation; CS = coronary sinus.

The ablation procedure was performed under mild sedation achieved with midazolam and fentanyl. These medications were administered by trained electrophysiology nursing staff under the supervision of doctors.^10^ The patient was in sinus rhythm at baseline. A 3-dimensional mapping system (EnSite X, Abbott) was used for ablation guidance and mapping. Bidirectional block across the cavotricuspid isthmus was confirmed by pacing maneuvers. After transseptal puncture was performed, completion of PVI and left atrial posterior wall isolation was confirmed by pacing maneuvers and 3-dimensional mapping with a high-density mapping catheter (Advisor HD Grid, Abbott). The left atrial anterior line was considered incomplete, and applications were delivered around Bachmann bundle using the PFA mode of the dual-energy, focal ablation catheter (TactiFlex Duo, Abbott), resulting in completion of the line. Subsequently, spontaneous atrial ectopy originating from the SVC was documented, prompting SVC isolation. After an activation map of the right atrium and SVC was obtained using the HD Grid (Figure 2A), applications were performed using the PFA mode of the TactiFlex Duo with normal waveform (2400 V) (Figure 2B).

Before each PFA application in the SVC, the right phrenic nerve was assessed by high-output pacing from the ablation catheter (20 mA, 2 ms), and sites demonstrating diaphragmatic capture were avoided during energy delivery. Continuous visual assessment of right diaphragmatic motion under fluoroscopy was performed during and immediately after each PFA application. During PFA application at the lateral aspect of the SVC (contact force 10–15 g), a sudden reduction in diaphragmatic excursion was observed (Supplemental Video 1A). Upon reassessment after approximately 3 minutes, diaphragmatic motion recovered (Supplemental Video 1B). Subsequent applications were performed using a low waveform (2100 V), resulting in successful SVC isolation with a total of 10 applications (contact force 10–15 g). After SVC isolation, remapping with the HD Grid catheter was performed (Figure 2C). At the end of the procedure, phrenic nerve pacing confirmed normal and symmetric diaphragmatic movement.

No other periprocedural complications occurred, and the patient was discharged on the second day of the procedure without symptoms or recurrence of atrial tachyarrhythmia. The 12-lead electrocardiogram at discharge is presented in Figure 1B.

The patient provided a written informed consent for the ablation procedure and agreed to the publication of her case details and images in this report.

## Discussion

This case illustrates that transient phrenic nerve stunning can occur during SVC isolation using a focal PFA catheter, despite assessment with high-output pacing before energy delivery, whereas previous reports of phrenic nerve dysfunction during PFA have primarily involved multielectrode or lattice-type PFA systems.

Unlike dedicated PFA systems using multielectrode or lattice-type catheters, the TactiFlex Duo delivers PFA through a focal ablation platform, potentially resulting in a distinct unipolar electric field geometry. The ability to switch between radiofrequency and PFA within a single catheter represents a significant technological advancement; however, it also necessitates careful evaluation of energy-specific safety considerations. Our observation suggests that, despite its favorable nonthermal profile, PFA delivered via this novel focal catheter can still transiently affect adjacent neural structures, particularly in regions with minimal spatial separation between myocardium and nerve tissue. Furthermore, a lower waveform (2100 V) was selected after the initial phrenic nerve stunning; however, the absence of a recurrent phenomenon should be interpreted with caution. The present case should not be interpreted as evidence that lower waveform settings are inherently safer for phrenic nerve preservation.

Several mechanisms may explain the observed transient phrenic nerve stunning. First, neural tissue has a higher threshold for irreversible electroporation than myocardium.^11^ Exposure to sublethal electric fields may result in transient membrane permeability changes, leading to temporary conduction block without axonal disruption. Second, PFA efficacy is determined by electric field distribution rather than direct tissue contact.^12^ In the anatomically constrained SVC region, the electric field may transiently affect the adjacent right phrenic nerve. Third, electroporation-related impairment of Schwann cell function may temporarily disrupt saltatory conduction, resulting in reversible diaphragmatic weakness.^13^ Fourth, close proximity of previous PFA may have caused unintentional stacking of PFA lesions, resulting in deeper lesions. Importantly, the rapid and complete recovery observed in this case supports a functional rather than structural nerve injury, consistent with transient neural stunning rather than permanent phrenic nerve palsy.

Several limitations should also be acknowledged in this report. First, no quantitative assessment of diaphragmatic motion was performed, and the diagnosis of phrenic nerve stunning was based on qualitative fluoroscopic imaging. Second, anatomic imaging for phrenic nerve visualization, which may provide additional information regarding phrenic nerve trajectory, was not performed in this case. Therefore, the exact spatial relationship between the ablation site and the phrenic nerve is insufficiently substantiated.

## Conclusion

Transient phrenic nerve stunning may occur during SVC isolation using the PFA mode of a dual-energy, focal ablation catheter. Although the phenomenon was reversible in this case, careful monitoring of phrenic nerve function during SVC ablation remains important to minimize the risk of persistent injury.

## Disclosures

C.E. has received research/travel grants and speaker honoraria from Abbott, Boston Scientific, LifeTech, Biosense Webster, CardioFocus, C.T.I. GmbH, and Doctrina Med. R.R.T. has received honoraria for lectures from Pfizer, Abbott, Biosense Webster, Boston Scientific, Doctrina Med, cme4u, Medtronic, Radcliff Cardiology, and Wikonect. He has received honoraria for advisory board participation and consulting from Boston Scientific, Biosense Webster, Capvision, Guidepoint, Haemonetics, Medtronic, Philips, and Abbott. His institution has received research funding or participated in clinical trials sponsored by Biotronik, Abbott, Boston Scientific, Medtronic, LifeTech, and Johnson & Johnson. He has also received travel grants from Biosense Webster, Abbott, Boston Scientific, Medtronic, and Philips. The other authors have no conflicts of interest to disclose.

## Footnotes

Appendix Supplementary data associated with this article can be found in the online version at https://doi.org/10.1016/j.hrcr.2026.04.018.
