# Supplementary material for: Transient phrenic nerve stunning during superior vena cava isolation after focal pulsed field ablation
Source: HeartRhythm Case Rep. 2026 Apr 20;12(7):713–6. doi: 10.1016/j.hrcr.2026.04.018 (PMC13379326; doi:10.1016/j.hrcr.2026.04.018)
Supplement: Supplementary Video Legends [file mmc2.docx]

**Video Legends**

**Video 1**

A: During PFA application at the lateral aspect of the SVC, a sudden reduction in diaphragmatic excursion was observed (40-degree left anterior oblique view).

B: Upon reassessment after approximately 3 minutes, diaphragmatic motion recovered (40-degree left anterior oblique view).

CS = coronary sinus; LAO = left anterior oblique.
